# Waitlisted and Transplant Patient Perspectives on Expanding Access to Deceased-Donor Kidney Transplant: A Qualitative Study

**DOI:** 10.1177/20543581221100291

**Published:** 2022-05-21

**Authors:** Canute Rosaasen, Nicola Rosaasen, Rahul Mainra, Aaron Trachtenberg, Julie Ho, Christina Parsons, Sean Delaney, Holly Mansell

**Affiliations:** 1 Johnson Shoyama Graduate School of Public Policy, University of Saskatchewan, Saskatoon, Canada; 2 Saskatchewan Transplant Program, Saskatchewan Health Authority, Saskatoon, SK, Canada; 3 Division of Nephrology, Department of Medicine, College of Medicine, University of Saskatchewan, Saskatoon, Canada; 4 Department of Internal Medicine, Max Rady College of Medicine, Rady Faculty of Health Sciences, University of Manitoba, Winnipeg, Canada; 5 Department of Internal Medicine and Department of Immunology, Max Rady College of Medicine, Rady Faculty of Health Sciences, University of Manitoba, Winnipeg, Canada; 6 Canadian Blood Services, Edmonton, AB, Canada; 7 College of Pharmacy and Nutrition, University of Saskatchewan, Saskatoon, Canada

**Keywords:** expanded criteria donor, marginal donor, kidney transplant

## Abstract

**Background:**

A concerning number of kidneys (eg, expanded donor criteria, extended criteria, or marginal kidneys) are discarded yearly while patients experience significant morbidity and mortality on the transplant waitlist. Novel solutions are needed to solve the shortage of kidneys available for transplant. Patient perceptions regarding the use of these less than ideal kidneys remain unexplored.

**Objective:**

To explore the perspectives of patients who have previously received a less than ideal kidney in the past and patients awaiting transplant who could potentially benefit from one.

**Design:**

Qualitative description study.

**Setting:**

2 provinces in Canada participated (Saskatchewan and Manitoba).

**Patients:**

Patients with end-stage kidney disease who were awaiting kidney transplant and were either (a) aged 65 years and older, or (b) 55 years and older with other medical conditions (eg, diabetes).

**Methods:**

Criterion sampling was used to identify participants. Semi-structured, one-on-one interviews were conducted virtually, which explored perceived quality of life, perceptions of less than ideal kidneys, risk tolerance for accepting one, and educational needs to make such a choice. The interviews were transcribed verbatim and thematic analysis was used to analyze the data.

**Results:**

15 interviews were conducted with usable data (n = 10 pretransplant; n = 5 posttransplant). Participants were a mean of 65.5 ± 8.8 years old. Four interrelated themes became prominent including (1) patient awareness and understanding of their situation or context, (2) a desire for information, (3) a desire for freedom from dialysis, and (4) trust. Subthemes of transparency, clarity, standardization, and autonomy were deemed important for participant education. The majority of pretransplant participants (n = 8/10) indicated that between 3 and 5 years off of dialysis would make the risk of accepting a less than ideal kidney feel worthwhile.

**Limitation:**

The study setting was limited to 2 Canadian provinces, which limits the generalizability. Furthermore, the participants were homogenous in demographics such as ethnicity.

**Conclusion:**

These findings indicate that patients are comfortable to accept a less than ideal kidney for transplant in situations where their autonomy is respected, they are provided clear, standardized, and transparent information, and when they trust their physician. These results will be used to inform the development of a new national registry for expanding access to deceased-donor kidney transplant.

**Trial Registration:**

Not registered.

## Abrégé

**Contexte:**

De nombreux reins sont rejetés chaque année (donneurs à critères élargis, critères étendus ou reins marginaux), alors que les patients qui attendent une greffe présentent une morbidité importante et un taux de mortalité élevé. De nouvelles solutions sont nécessaires pour contrer la pénurie de reins disponibles pour une transplantation. Les perceptions des patients quant à l’utilization de ces reins moins idéaux restent inexplorées.

**Objectif:**

Explorer les perceptions des patients ayant reçu un rein moins idéal dans le passé et des patients en attente d’une greffe qui pourraient potentiellement bénéficier d’un tel don.

**Conception:**

Étude qualitative et descriptive.

**Cadre:**

Deux provinces canadiennes (Saskatchewan et Manitoba).

**Participants:**

Des patients atteints d’insuffisance rénale terminale en attente d’une transplantation (a) âgés de 65 ans et plus ou (b) âgés de 55 ans et plus et présentant d’autres problèmes de santé (ex. diabète).

**Méthodologie:**

L’échantillonnage avec critères a été utilisé pour identifier les participants. Des entretiens individuels semi-structurés menés virtuellement ont exploré la qualité de vie perçue, la perception quant aux reins moins idéaux, la tolérance à l’égard des risques inhérents à l’acceptation d’un tel rein, et les besoins d’information pour faire ce choix. Les entrevues ont été transcrites intégralement et l’analyze des données a été réalisée par analyze thématique.

**Résultats:**

Quinze entrevues avec données utilisables ont été menées (n = 10 avant la greffe; n = 5 après la greffe). Les participants avaient en moyenne 65.5 ± 8.8 ans. Quatre thèmes interreliés ont été dégagés : (1) la sensibilisation et la compréhension des patients quant à leur situation ou au contexte; (2) le besoin d’information; (3) le besoin d’un congé de dialyze; et (4) la confiance envers le médecin. La transparence, la clarté, la normalization et l’autonomie ont été jugées comme des sous-thèmes importants de l’éducation des participants. Pour la majorité des participants en attente d’une greffe (n = 8/10), l’idée d’un congé de 3 à 5 ans de dialyze rendrait acceptables les risques associés à l’acceptation d’un rein moins idéal.

**Limites:**

Étude tenue dans deux provinces canadiennes, ce qui limite la généralisabilité des résultats. Homogénéité des participants sur le plan démographique, notamment en ce qui concerne l’origine ethnique.

**Conclusion:**

Les résultats indiquent que les patients seraient à l’aise d’accepter un rein moins idéal pour une greffe, pourvu que leur autonomie soit respectée, qu’ils reçoivent des informations claires, standardisées et transparentes, et qu’ils aient confiance en leur médecin. Ces résultats serviront à éclairer l’élaboration d’un nouveau registre national afin d’élargir l’accès à la transplantation de rein provenant de donneurs décédés.

**Enregistrement de l’essai:**

Non enregistré.

## Introduction

Kidney transplantation has revolutionized the treatment of end-stage kidney disease; however, the shortage of donor kidneys remains a critical barrier. In 2019, more than 3000 Canadians were on the kidney transplant waitlist, but only 54% received a transplant.^1^ While aggregate statistics on discarded kidneys are not available in Canada, data indicate that nearly 1 in 5 kidneys available for transplant in the United States are discarded.^2^ Novel efforts for optimizing organ use are urgently needed to mitigate this mismatch.

Kidneys are discarded for a myriad of reasons, with 2 of the top predictive factors being donor age and creatinine.^3^ Kidneys retrieved from expanded criteria donors (ECDs), defined as those obtained from deceased donors ≥ 60 years, or donors aged 50-59 years with 2 of following: cerebrovascular cause of death, terminal serum creatinine > 132.6 μmol/L (1.5 mg/dL), or history of hypertension,^4^ are more likely to be discarded.^3,5,6^ In an analysis of registry data (October 1999 to June 2005) nearly half (41%, 5139/12 536) of ECD kidneys were not used.^5^ Further work has highlighted a significant variation in acceptance rates of lower quality organs between settings, suggesting that kidneys considered unusable by 1 transplant center, could be acceptable for another.^6,7^ Potential wastage must be critically examined, as many patients may derive a survival benefit from an ECD kidney transplant, when compared with remaining on dialysis while awaiting a better organ offer.^8,9^ Less-than-ideal (LTI) kidneys (eg, expanded donor criteria, extended criteria or marginal) may be of particular benefit for those who are older or at decreased likelihood of receiving a kidney.

Canadian Blood Services (CBS) is a not-for-profit organization that collaborates with the Organ and Tissue Donation & Transplantation (OTDT) community to improve national system performance, including managing clinical programs that support interprovincial sharing of organs.^10^ CBS is exploring a national registry for patients who may be interested in receiving a transplant that is considered LTI but acceptable. A registry could optimize the use of deceased-donor kidneys by standardizing practice, providing a mechanism for offering/sharing organs, enhancing transparency and efficiency, and offering new transplant opportunities for dialysis patients.

Although patient-centered priorities are key for successful implementation of health care interventions,^11^ there is a lack of data describing what Canadians with kidney disease think about LTI kidneys. Previous studies have investigated transplant candidates’ understanding and preferences on increased risk/marginal donors,^12-16^ but use of LTI kidneys remains inconsistent and unexplored. A recent qualitative study in the United States explored perspectives on high kidney donor profile index (KDPI) kidneys (KDPI > 85).^17^ Interviews were conducted with clinicians (surgeons, nephrologists, and nurse coordinators), and patients (pretransplant and posttransplant) to characterize experiences on consent, education, and the decision-making process. A local study on LTI kidneys is necessary to underpin a national initiative to maximize and standardize use in Canada, with particular focus on patients who may be interested in such a registry. The purpose of this study was to specifically explore the perspectives of those who have previously received an LTI kidney and those who may benefit from this type of transplant. We also sought feedback on what education would be required to support an informed decision to accept an LTI kidney.

## Materials and Methods

A qualitative description^18^ study was undertaken in 2 Canadian provinces (Saskatchewan and Manitoba). This study was approved by the University of Saskatchewan Behavioural Ethics Board and all participants provided informed consent (Beh #2764).

### Participants and Recruitment

To explore the perspectives of those who may benefit from an LTI transplant, we included patients with end-stage kidney disease who were awaiting kidney transplant and were either (1) aged 65 and older, or (2) 55 and older with other medical conditions (eg, diabetes). These parameters were chosen as they best represented the perceptions of the population of interest (individuals who would be in a situation to consider an LTI kidney registry). We also included patients who had received a kidney transplant which was described by their physician as ECD (or synonymous terms), to learn what patients would have liked to know with the benefit of hindsight. Criterion sampling^19^ was used, whereby potential participants were identified by transplant nephrologists at the Saskatchewan Transplant Program and Transplant Manitoba and provided with an invitation letter. A research team member contacted patients who expressed interest in learning more about the study.

### Data Collection

A semistructured interview guide was created by 4 members of the research team (C.R., H.M., N.R., and R.M.; a graduate student in public policy, health science researcher, pharmacist, and nephrologist, respectively). It was reviewed by the rest of the research team and the CBS steering committee (consisting of a transplant recipient, 13 transplant nephrologists, surgeons, and Organ Donation Organization [ODO] executives representing various regions in Canada). The interview guide, which was inductive in nature (Supplemental Appendix A) consisted of exploratory questions pertaining to 4 domains: perceived quality of life (QOL), perceptions of LTI kidneys, risk tolerance for accepting an LTI kidney, and educational needs. Questions to the pretransplant cohort were posed to evaluate perspectives on their condition, perceptions of LTI kidneys, transparency of the process and to speculate and evaluate how a hypothetical kidney may benefit them. To investigate risk tolerance, a case-based scenario was used.^20,21^ The posttransplant group was asked about their current perspective on their health status and the impact of their transplant, LTI kidneys in general, transparency of the process in relation to LTI kidneys, and to speculate on how a hypothetical kidney may serve someone other than themselves. Self-reported demographic data including mode of dialysis, residence location, gender, marital status, education, and ethnicity were collected. The one-on-one (1 time) interviews were conducted virtually using the Webex Cisco platform. A University of Saskatchewan (male) graduate student in public policy with experience in performing interviews, and not known to the participants (C.R.), conducted the meetings. The student, who did not have a background in transplantation, came to the project with an interest in deliberative dialogue and participatory policy-making. Field notes were made during the interview to provide context to the data. An honorarium of CAD $75 was provided to participants.

### Data Analysis

Interviews were transcribed by the Canadian Research Hub and anonymized. Transcriptions were input into NVivo12 (QRS International Pty Ltd, 2021) and reflexive thematic analysis, as described by Braun and Clarke,^22,23^ was used to analyze the data. C.R. (who had previous experience in qualitative analysis) created the initial codes and managed the data set. H.M., N.R., and C.R. met on multiple occasions allowing for reflection, discussion, code, domain, and theme relabelling, and refinement. During the first phase and second phase (familiarization with the data and generating initial codes), the transcripts were read multiple times, and the audiotapes were reviewed as needed to verify the data. A variety of semantic and latent codes were applied through the first pass through the data, at which point it was determined that a code book would be useful for organizing the semantic codes according to domains. To manage the varying perspectives (prospective vs retrospective), the pretransplant and posttransplant groups were initially coded separately. During phase 3 (generating themes), codes were collapsed into draft themes and subthemes. In phase 4 (reviewing potential themes), the themes were reviewed within each cohort (pretransplant vs posttransplant), and across the data set. During phase 5 (defining and naming them), the themes and subthemes were reviewed for internal homogeneity and external heterogeneity and related back to the research question.^24^ During phase 6, the report was produced and sent to the participants who were asked whether there was any additional information that should be added.

## Results

The research associate contacted the 37 patients that expressed an interested in learning more about the study. Of them 16 agreed to be interviewed (n = 11 pretransplant, n = 5 posttransplant), but 1 pretransplant participant’s audiotape was inaudible and therefore 10 were used in the analysis. Of those who did not participate, patients either did not answer the phone or declined. The reasons for not participating were not explored in further detail. After approximately 9 interviews, the content seemed to be largely repetitive and the key themes that emerged were reinforced through the subsequent interviews. After member checking, some participants reiterated existing information (ie, that increasing the number of kidneys available is a positive goal and/or that they disliked dialysis) but no information was volunteered that changed the interpretation of the data or the resulting report. The interviews lasted between 15 and 35 minutes (mean 26:34, median 28:54). The participants ranged in age from 55 to 76 years and majority (73%) were male. Tables 1 and 2 describe the self-reported patient demographics for the pretransplant and posttransplant cohorts.

**Table 1.** Self-Reported Demographics of the Pretransplant Participants, n = 10.

Pretransplant participant characteristics	
Age	65.5 ± 8.8 (range 55-76 years)
Gender (male)	7 (70)
Ethnicity (as reported by participant)	
Canadian	1 (10)
Caucasian	7 (70)
Filipino	1 (10)
East Indian	1 (10)
Marital status	
Married/common-law/widowed	7 (70)/1 (10)/2 (20)
Education, highest achieved	
Grade 10/High school/Postsecondary	1 (10)/5 (50)/4 (40)
Residence	
Urban/Rural/Unknown	6 (60)/3 (30)/1 (10)
Prior transplant	
No/Yes/Unknown	8 (80)/1 (10)/1 (10)
Wait time (years)	4.9 ± 2.4 (range 1-7 years)

Data are reported as *M* ± *SD* or counts (%).

**Table 2.** Self-Reported Demographics of the Posttransplant Participants, n = 5.

Posttransplant participant characteristics	
Age	66.6 ± 5.7 (range 60-74 years)
Gender (male)	4 (80)
Ethnicity (as reported by participants)	
Canadian	1 (20)
Caucasian	3 (60)
Métis	1 (20)
Marital status	
Married	5 (100)
Education, highest achieved	
High school/Postsecondary	1 (20)/4 (80)
Residence	
Urban/Rural	2 (40)/3 (60)
Prior transplant	
No/Yes	3 (60)/2 (40)
Time since transplant (years)	2.6 ± 2.5 (range 7 months to 7 years)

Data are reported as *M* ± *SD* or counts (%).

The pretransplant and posttransplant participants were asked to rate their QOL on a Likert-type scale of 1-5 (1 = *very poor*, 5 = *very good*), and to describe the reason for their QOL rating. To obtain a sense of impact of disease, posttransplant participants were also asked to indicate how their transplant has affected their QOL on a scale of 1-5 (1 = *much worse*, 3 = *the same as before*, 5 = *much better*) (Tables 3 and 4).

**Table 3.** Perceived QOL by Participants—Posttransplant, n = 5.

QOL^a^ (day of interview)	Change in QOL due to transplant^b^	Reasons cited
4	5	Energy, convenience, return to normalcy, freedom
3	4	Energy, convenience, return to normalcy, freedom
4.5	5	Convenience, health benefit, return to normalcy
5	4	Convenience, freedom
5	5	Energy, convenience, health benefit

*Note.* QOL = quality of life.

a Participants were asked to rate their QOL on a Likert scale of 1-5 (1 = *very poor*, 5 = *very good*).

b Posttransplant participants were asked to specify how transplant has affected their QOL on a scale of 1-5 (1 = *much worse*, 2 = *worse*, 3 = *the same as before*, 4 = *better*, 5 = *much better*). (QOL mean 4.3 ± 0.8, range 3-5; Change QOL 4.6 ± 0.5, range 4-5.)

**Table 4.** Perceived QOL by Participants—Pretransplant, n = 10.

QOL^a^ (day of interview)	Reasons cited
4	Although inconvenient, dialysis has become part of routine
3	Inconvenience, lack of energy, lifestyle changes
5	No noticeable impact
4	Inconvenience, physical discomfort, lifestyle changes
3	Unable to work, lack of energy
3	Lifestyle changes, lack of energy
3	Unable to work, lifestyle changes, lack of energy, physical discomfort
3	Lifestyle changes, physical discomfort
3	Lifestyle changes, lack of energy
4	No noticeable impact pre-disease to dialysis, inconvenience

*Note.* QOL = quality of life.

a Participants were asked to rate their QOL on a Likert scale of 1-5 (1 = *very poor*, 5 = *very good*). (QOL mean 3.5 ± 0.7, range 3-5.)

**Figure 1.**
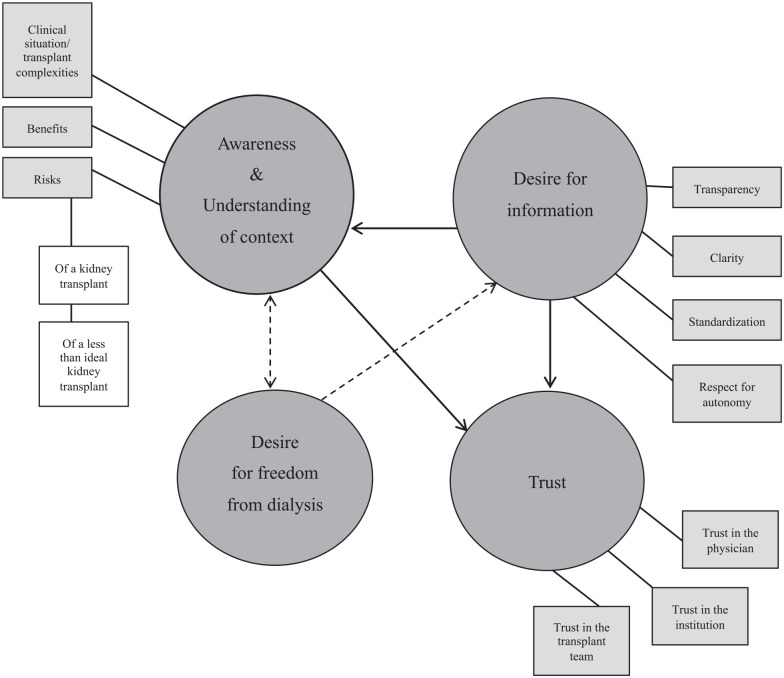
Themes and subthemes identified from the interviews. The circles represent the themes while the gray rectangles represent the subthemes. The arrows illustrate how the themes are interrelated.

Four overarching themes became prominent after the data were analyzed. These included (1) patient awareness and understanding of their situation or context, (2) a desire for information, (3) a desire for freedom from dialysis, and (4) trust. These themes were consistent amongst both the pretransplant and posttransplant participants. The posttransplant cohort had the benefit of hindsight, and reinforced (with more confidence) the sentiments expressed by the pretransplant cohort. The posttransplant cohort also had firm interpretations on their improvement to QOL. Figure 1 depicts the themes and how they relate are interrelated: The desire for freedom from dialysis illustrates patient awareness of their clinical situation and provides context to the benefits and risks of accepting an LTI kidney. To further contextualize their clinical situation, patients want information that is clear, transparent, and standardized. Information that is presented in this way enhances patient understanding of their clinical situation while supporting the development of trust in their physician and team. Table 5 provides additional supporting quotes.

**Table 5.** Themes, Subthemes, and Additional Supporting Quotes.

Theme	Subtheme	Quote
Theme 1: Awareness and Understanding of Context		Participant 2 (posttransplant): “Well, the risks are that it never works.” Participant 11 (pretransplant): “I know it’s like a crap shoot most of the time, but you just never know. It could last longer than expected or shorter than expected.” Participant 6 (pretransplant): “You know what, in the meantime, less than ideal kidneys, you know, there’s a market for it. There’s a demand for it and I’m sure there’s lots of recipients that would be more than happy to accept that compared to the alternatives.” Participant 5 (posttransplant): “at my point in life I think having a kidney that works even if there are a few implications, I still think it was the right thing to do.” Participant 8 (posttransplant): “Well, I guess you’d have to gaze deeply into a crystal ball and predict how long it’s gonna last.” Participant 9 (pretransplant): “You could die in surgery or something like that. That’s the risk you’re taking, but that’s acceptable.” Participant 11 (pretransplant): “You know, for me, a case-by-case basis. If I was offered a LDI kidney, I would look at it as let’s say it was a Hepatitis C kidney, so I could say to myself ‘well we can see if we can manage that. How long will it give me?’ Who knows? It all depends. Personally, I would probably say yes, I’ll go for it.”
Theme 2: Desire for information	Transparency	Participant 11 (pretransplant): “Is it from an older patient? Is it hepatitis C? Is it whatever for whatever reason? So I just have an idea of what I’m getting into. . . Why is it less than ideal and how we can mitigate some of those issues? A ballpark longevity of the kidney would be nice.” Participant 7 (pretransplant): “I would have to know the medical reasons why the kidney is not 100% viable. I would have to look at what the terms are that the person had who was giving up the kidney, just exactly how that would impact my life. Like was it a diabetic? Was it somebody that had some other issues, cancer or whatever?” Participant 16 (pretransplant): “First of all, I would like to know how many times we have to go for checkup after it’s been transplant and what kind of medications that we have to take or antibiotics. And what kind of precautions we have to keep in our head and diet-wise, exercise wise or what can we do and what we can’t.” Participant 10 (pretransplant): “I guess I would like to look at everything they had and maybe success rates of previous similar transplants.” Participant 12 (pretransplant): “I’d appreciate if they would tell me the risks and how long that they predict that this thing might last or if it’ll last until I die, who knows?”
	Clarity	Participant 3 (posttransplant): “I’d just like a better explanation of what less than ideal means. I guess I should do the research now but I don’t know what all that involves” Participant 8 (posttransplant): “But also you need to understand why it’s marginal and once you understand why it is marginal, then you need to be able to evaluate if, excuse the expression, marginality will be a big detriment to your health or not.” Participant 1 (pretransplant): “Well I did have a conversation with my transplant doctor and he sort of explained this less than perfect kidney, too. And I thought well it’s kind of encouraging in a way even though it may not last as long as a young kidney.”
	Standardization	Participant 3 (posttransplant): “You know, I know if somebody would’ve explained this to me months or whatever before the transplant, I would’ve liked to read up on it and see what it’s all about. I think I could’ve made a more informed decision. I’m sure my decision would’ve been the same though.” Participant 8 (posttransplant): “You can’t expect somebody to make decisions at 3:00 in the morning and you got a kidney there waiting to be transplanted. It’s too hard. It all has to be done preemptively.”
	Autonomy	Participant 1 (pretransplant): “I think they should give us the choice.” Participant 2 (posttransplant):”I still think they should have a choice.” Participant 11 (pretransplant): “I would probably have to with some discussion there. With the patient and together the doc, and the patient should decide if this is something acceptable for that individual.” Participant 14 (pretransplant):”But as a transplant specialist, you’re taking that chance away from them whether you realize it or not.” Participant 14 (pretransplant): “Well. I think whoever’s a good match for it should get it, should have that right to decide if they want it. You know?” Participant 2 (posttransplant): “But he gave me the choice which was the big thing.” Participant 3 (posttransplant):”I mean it’s an individual choice and that’s fine. For me, it seemed like a risk I was willing to take.”
Theme 3: Desire for freedom from dialysis		Participant 10 (pretransplant): “Well I guess just getting a kidney at all is a plus. And reason being maybe just to get some good years in before I’m a senior.” Participant 2 (posttransplant): “When I was on dialysis, I lived out of town, so I’d have to hop the bus, and it would be a full day of travel, dialysis, and then come home again. Before dialysis, you feel lousy, after dialysis, you feel great, and then the next day, you’re lousy again. And now, I feel perfect. I can get out and do things. I’ve got a life. . .Whatever it gives you, it’s better than nothing.” Participant 12 (pretransplant): “I’m talking about if I got a kidney today, it’d be a life changing situation for me. I could probably eventually do a little more. I could get out golfing like I used to and maybe walk a little better. You know, do some things that I don’t even do anymore cause I just sit here at home most the time after dialysis and read the paper, watch tv. I’d like to go play poker with the guys on Saturday night, stuff like that.”
Theme 4: Trust		Participant 12 (pretransplant): “He would have the more information on whether it would be good for me or not cause I’m a lay person in this outfit and I usually go by what the doctors say, not what I say” Participant 3 (posttransplant): “I also accept that these people make their judgment based on the education they have, the experience they, which is by far more than a layman would have and I do trust that the doctor’s judgment would be made in a fair and sort of equitable manner.” Participant 9 (pretransplant): “Well, I think I wouldn’t make that decision by myself, I’ll tell you that. I think if the doctor isn’t willing to make that decision, then it’s all left up to me, I don’t need a doctor then, do I?”

### Theme 1: Awareness and Understanding of Context

Participants in this study expressed an awareness and understanding of their clinical situation and complexities of the transplant process, as well as the potential benefits and risks of (a) accepting a transplant and (b) accepting an LTI kidney. The benefits of accepting any type of transplant included freedom from dialysis (cited 17 times, pretransplant cohort), the ability to travel (cited 9 times), QOL improvement (7), health and life expectancy benefits (5), and a perceived return to normalcy (11). Risks of remaining on dialysis included associated illness (cited 10 times), lack of energy (14), inability to undertake activities, or lack of freedom (10).

The risks of accepting a transplant were referenced 16 times throughout the data without query, and the risks of accepting an LTI kidney were mentioned 28 times with prompting. The potential for decreased longevity with an LTI kidney was the most concerning risk, accounting for 18 of the 28 references. According to participant 7 (pretransplant),

It[the risk]’s more centered on how long it would last than how effective it would work. . .I mean you’re not gonna go through all of that surgery and recovery and all of that and then only have six months. It’s not really worth it to go through all that.

One consistent trend within this theme was that patients were aware and accepting of the risks associated with LTI kidney transplant. Many patients articulated risks associated with transplant (such as rejection, death, significant recovery time, surgical risks, and risks of increased medication) and proceeded to engage rationally with that risk. Participant 3 (posttransplant): “I’d say there’s risks, there’s also benefits. You have to decide if you want to accept the risk and if somebody doesn’t—I mean it’s an individual choice—and that’s fine.”

Participants also articulated an understanding of scarcity of kidneys for transplant and a reasonable understanding of the challenges of allocation. Interestingly, patients seemed to evaluate the risk of transplantation in reference to their age and scarcity of resource suggesting an understanding and calculation about how they would like to spend the “time they had left.”

Participant 6 (pretransplant) said, “Well, people are dying waiting for an optimal kidney, and if this has a possibility of extending their lifespan even though it may not be an ideal kidney, compared to death, some quality of life would be of benefit.”

### Risk Tolerance Scenario

Using the case-based scenario (supplementary materials), participants in the pretransplant group were asked how many dialysis free years would make the risk of accepting an LTI kidney feel worthwhile. The majority of participants (n = 8/10), responded with a number between 3 and 5 years, while 1 participant responded with a year or less. An outlier (participant 6) responded with 15 years. According to participant 12 (pretransplant): “In my case, I’m 74. If it gave me—I don’t know—anywhere from say without any other problems, five to ten years of more life might be acceptable, right?” The posttransplant recipients were instead asked whether they would make the same decision to accept an LTI kidney. Four out of 5 participants said yes and indicated they would sign up for such a registry.

### Theme 2: Desire for Information

Patients articulated a desire for information to support informed decision-making about LTI kidneys, which were evident in the 4 subthemes.

#### Transparency

Patients indicated that when kidneys that are offered to them, they wanted the information to be presented honestly and without obfuscation about the quality and prospects of the kidney. There was a sense that information needs to be presented in plain language that is not open for varied interpretation. Having said that, some participants mentioned that kidney offers come with an emotional cost to patients and that, while information should be transparent, it should be presented with empathy. According to participant 8 (posttransplant), “You have to remember there’s a whole element of emotional turmoil associated with getting ones hopes elevated and then bashed. That’s a big issue.”

#### Clarity

Patients expressed a desire for the medical system to express, in clear and understandable terms, both the quality of kidneys presented to them, as well as their expected outcomes in receiving a transplant. Historically, terms such as ECD or marginal have been used as descriptors. When asked what patients perceived to be most appropriate for this type of kidney, clear terminology such as “less than ideal” was preferred by 8 of 15 participants. According to participant 11 (pretransplant), “It’s straightforward, it’s not wishy washy with fancy words.”

#### Standardization

Patients indicated a need for standardized education about the various types of kidneys, and potential outcomes associated with that choice. They articulated a need for this information to be presented to them during the assessment process. They recognized that they may be on the waitlist for significant periods of time, and they recommended that terminology remain consistent throughout the assessment process, waitlist, and transplant process. There was the sense that this standardization would contribute to patient comprehension, which also underpins both autonomy and trust in their physician and health care system.

In addition to the desire for standardization from a “personal journey” perspective, uniformity was also perceived to be important for the registry, with consistent definitions and criteria for offers applied nationally. One of the interview guide questions asked participants to consider a scenario where an LTI kidney offer was declined by 1 program, only to be utilized for transplant by another. One participant expressed concern that lack of standardized criteria could lead to a missed opportunity. Participant 1 (pretransplant), “Especially if they turn around and offer it [the kidney] to somebody else.” This can be interpreted as a desire for national standardization to ensure that local standards do not result in missed opportunities.

#### Autonomy

Several (8) participants expressed a desire for their personal health care decisions to be respected. This concern for autonomy was clear but qualified with a desire to be equipped with appropriate and understandable information on which to base personal health decision-making. Participant 8 (posttransplant) stated, “I think it [education about LTI kidneys] should all be done beforehand.”

### Theme 3: Desire for Freedom From Dialysis

Participants in the pretransplant and posttransplant cohort perceived transplantation as the ultimate solution to become free from dialysis. Participant 8 (posttransplant) stated, “I just kind of want some relief and get the hell off of dialysis.” Even though most participants were of retirement age, they regarded dialysis as a significant prohibition to activities that would improve their quality of life, such as traveling, visiting with grandchildren, or pursuing personal interests. According to Participant 3 (posttransplant), “It just ties up your life so much. It’s like you’re existing, you’re not really living.” Participant 14 (pretransplant) said, “Now that I’m stuck this in place, I think even if you had the opportunity to have a kidney that was 50% or 60%, it’s better than no kidney.”

### Theme 4: Trust

The final theme explicated in this study was sense of trust, which was consistent in the interviews but not universal. Of the 16 participants, 12 patients made an *overt* mention of their trust in their physicians and the medical institution. This was consistent throughout both the pretransplant and posttransplant groups. In this context, we note institution in reference to “the medical system”—many patients stated or implied that they trust that organs will be distributed in a fair and equitable manner that maximizes utility. Notably, the posttransplant group were more inclined to highlight their trust in their transplant *team*, in addition to their physician.

The majority of patients expressed that they generally trusted doctors to both screen out kidneys that were inappropriate for them and guide them through evaluating kidneys that the physician’s deemed viable. According to participant 9 (posttransplant), “No, I don’t need a guarantee, what I need is an opinion and that’s why I have a doctor is because I trust them.” Participant 9 (pretransplant) stated,

I would probably leave it in the hands of the medical professionals as to what would be considered a viable kidney to transplant or not. I think if it was up to the patients or if they had some input, there might be some people willing to accept basically any kidney at all and I think a medical professional would be in a better position to determine what would be suitable and appropriate than a patient.

## Discussion

Four interrelated themes describe transplant patient perspectives on expanding access to deceased-donor kidneys. First, participants acknowledged that organs for transplant are a scarce resource and an understanding of how their age will affect their opportunity to receive and benefit from a kidney transplant *(theme 1: patient awareness and understanding of context)*. The risks associated with surgery, immunosuppression, and recovery time were cited frequently, suggesting an awareness of the ongoing care required for older individuals, while balancing the desire for freedom from dialysis even though there may be a shortened duration of benefit. Of note, we anticipated the participants to be tentative in explaining their opinions on the novel concept of accepting an LTI kidneys and had expected this topic would require explanation and discussion. Participants, however, were decisive and confident in their responses; on average the interviews took about 25 minutes because participants were able to articulate these opinions clearly and succinctly.

Patients were able to use their comprehension to assess a cost-benefit analysis for accepting an LTI kidney, and most participants indicated they would accept an LTI kidney now if it gave them 3-5 years of freedom from dialysis, while accepting that long-term graft survival may be reduced. This is in line with other work showing that patients are willing to accept higher risk organs, particularly when their clinical situation is deteriorating.^14,17,21^ Participants also articulated a desire for freedom from dialysis citing disadvantages such as the inability to travel or pursuing personal interests which would improve their quality of life *(theme 3: desire for freedom from dialysis).* Given that our study population was older with comorbidities, this finding is noteworthy, as it indicates that the dialysis-associated costs to these patients could be as significant as the inability to participate in the economy or socialize might be to a younger patient. It also supports the use of LTI kidneys for transplant to facilitate freedom from dialysis for patients in older age groups.

Valuable insights were garnered on the type of education required for patients considering an LTI transplant (*theme 2: desire for information).* Participants repeatedly indicated that education presented with transparency, clarity, and standardization was important to them. Information provided in this way contributes to trust in the health care system *(theme 4: trust)* while retaining the autonomy to make supported choices about their health *(theme 2: desire for information).* In our study, as in others, patients had a strong desire to be involved in the decision-making process.^15,25^ Standardization emerged as a theme to ensure an “equal footing” between patients within a program as well as nationally to ensure that local opportunities are not missed. This suggests that the proposed national program should consider the application of standardized terminology and teaching to allow for informed decision-making by both health care providers and patients. Participants require such education both during the assessment period and when they are offered a kidney. During the time of the offer, they would appreciate information about the nature of the kidney being offered and the anticipated clinical course from accepting the organ. Like other work, participants indicated that specific features about the donor and the plausible risks may impact their choice,^12,20,26^ and that they generally trusted doctors to screen out kidneys that were inappropriate for them and guide them through the decision-making process.^12^ This suggests that while patients desire information and agency in their interaction with the medical system, special attention and careful dialogue is preferable from a patient perspective; thoughtless interaction with patients can lead to significant disappointment and emotional turmoil.

The limitations of this study should be considered. First, qualitative methodology by nature is subjective and can be subject to the preconceptions of the interviewer/researcher. We attempted to minimize bias by having a graduate student in public policy conduct the interviews and perform the coding rather than a transplant practitioner or researcher who would have their own preconceived notions. From an educational perspective, we felt it was important to include posttransplant patients in this study, to learn whether they felt prepared and what information was perceived to be lacking. Including these participants, however, could have skewed the perceptions since their circumstances likely led to a positive outcome. The study setting was limited to 2 Canadian provinces, which limits the generalizability. Furthermore, the participants were homogenous in demographics such as ethnicity which is a significant limitation. This sample was representative of the patients who were cared for by the (Saskatoon) Saskatchewan Transplant Program at the time (where 1/26 individuals on the waitlist, 1/8 patients completing assessment, and 5/24 posttransplant recipients met the inclusion criteria and self-identified as Indigenous). A future study, however, is needed to explicitly explore the perspectives of patients who identify as First Nations or Métis, or other populations who may be under-represented on the waitlist, or who may have different perspectives based on varying levels of trust of the health care system. While this study provided insights from Canadian patients on LTI kidneys, it was only was just a starting point. Further consultations are needed to guide the development of a new registry, such as delving further into specifics about the ideal timing of the education, and patient understanding of risks and benefits of receiving an LTI kidney.

## Conclusion

Participants in this study exhibited a self-awareness and understanding of their clinical situation and context and were able to articulate a risk-benefit calculation for accepting an LTI transplant. In order to feel most comfortable with decision-making, they require clear and digestible education that remains consistent until the time of their transplant offer. They need their education and transplant offers to be honest and transparent to allow them to exercise their autonomy while maintaining trust in their physician and the system. When these conditions exist, patients are willing to accept LTI kidneys to obtain freedom from dialysis. Transparency, clarity, and consistency, and respect for autonomy should be key tenets in the movement toward more successful use of LTI kidneys.

## Supplemental Material

sj-docx-1-cjk-10.1177_20543581221100291 – Supplemental material for Waitlisted and Transplant Patient Perspectives on Expanding Access to Deceased-Donor Kidney Transplant: A Qualitative Study Click here for additional data file. Supplemental material, sj-docx-1-cjk-10.1177_20543581221100291 for Waitlisted and Transplant Patient Perspectives on Expanding Access to Deceased-Donor Kidney Transplant: A Qualitative Study by Canute Rosaasen, Nicola Rosaasen, Rahul Mainra, Aaron Trachtenberg, Julie Ho, Christina Parsons, Sean Delaney and Holly Mansell in Canadian Journal of Kidney Health and Disease
