# Supplementary material for: Waitlisted and Transplant Patient Perspectives on Expanding Access to Deceased-Donor Kidney Transplant: A Qualitative Study
Source: Can J Kidney Health Dis. 2022 May 21;9:20543581221100291. doi: 10.1177/20543581221100291 (PMC9125065; doi:10.1177/20543581221100291)
Supplement: sj-docx-1-cjk-10.1177_20543581221100291 – Supplemental material for Waitlisted and Transplant Patient Perspectives on Expanding Access to Deceased-Donor Kidney Transplant: A Qualitative Study [file sj-docx-1-cjk-10.1177_20543581221100291.docx]

**Appendix A. Semi-structured interview guide**

|  | **Questions asked to both cohorts** | **Waitlisted patients** | **Post-transplant patients** |
| --- | --- | --- | --- |
| **Introductory** | Where are you on your transplant journey?  What do you know about the role of CBS in developing national kidney sharing programs? |  |  |
|  | **Section 1: QOL Assessment**  How would you rate your quality of life? (1=very poor, 2=poor, 3=neither poor nor good, 4=good, 5=very good)  Why did you choose the number you did? | Compared to before you had kidney disease, how has your quality of life changed on a scale of 1-5? (1=much worse, 3=the same as before, 5=much better) | Compared to before you had your kidney transplant, how has your quality of life changed, on a scale of 1-5? (1=much worse, 3=the same as before, 5=much better) |
|  | **Section 2: Perceptions**  What do you perceive as benefits of accepting a less than ideal kidney?  What do you perceive as risks of accepting a less than ideal kidney?  What do you perceive as benefits of not accepting a less than ideal kidney?  What do you perceive as risks of not accepting a less than ideal kidney? |  |  |
|  | **Section 3: Risk Tolerance** | **PREFACE:** For the next section, we would like you to consider the following scenario:   - Imagine you were offered a less than ideal kidney for transplant. - Your transplant doctor tells you that although the kidney is less than ideal, it is a good match for you and could offer you freedom from dialysis (for a few years). - It is possible that this kidney may not work immediately, and you may need dialysis (for 2-3 weeks) before it starts working. - If you accept this kidney, you may need more medical care for the time that it works compared to a transplant with a (“better”) kidney. - Your transplant doctor also tells you that there is no guarantee that another or better kidney will become available for you if you decide to decline this offer.   How many years off dialysis would you need to make this a good choice for you? (Please explain) | Thinking of your own circumstance, would you say that the benefits to your health and quality of life outweighed the risk you took accepting a less than ideal kidney?  (Please explain)    -What has been the best thing about receiving your kidney transplant?  -What has been the worst thing about receiving your kidney transplant?  -If you had to make the choice again about accepting a less than ideal kidney, would you make the same decision?  (Please explain) |
| **Exploratory Questions** | **Section 4: Education**  What information would you want from your kidney doctor that would allow you to decide to accept a less than ideal kidney?  What information would you want before signing up for this potential registry?  PREFACE: In this question we will go through a scenario about less than ideal kidneys.   - When a less than ideal kidney is offered to your transplant program, the transplant doctor on call uses their professional judgement to decide whether this kidney would benefit anyone on their provincial waitlist. - If they do not feel it would offer a benefit, the transplant doctor declines the kidney offer. - In this scenario, the decision made by your transplant doctor was made without the input of any patients on the waitlist. - The kidney is then offered to other provincial transplant programs in the country because another transplant doctor may have a different opinion about the kidney. It is possible that this kidney would then be transplanted. - If the kidney is not accepted by any programs, it would be discarded.   Do you think the doctor should first communicate the offer with a patient before turning it down, OR do you think the doctor should “screen out” some kidneys without discussion?  We have used term less than ideal throughout this interview. Other terms that have been used to describe this kind of kidney are marginal, extended criteria, medically complex donor. Is there another term that be better?  There has been a bit of debate about what to call this registry. (Extended recipient pool has been suggested) What do you think would be an appropriate title? |  | If you were asked for advice from a person trying to decide whether to accept a less than ideal kidney, what would you tell that person?  PROBES:  *Was there anything that happened related to your transplant that you didn’t expect?* |
| **Exit** | Is there anything else that you would like to tell us about that you think is important? |  |  |
